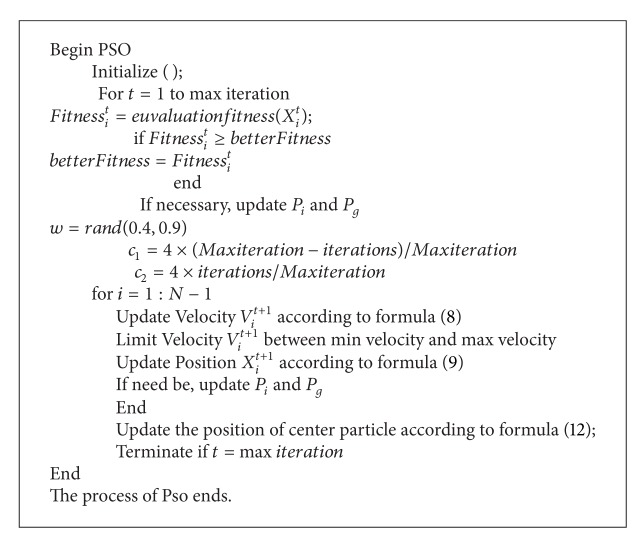# Multiple R&D Projects Scheduling Optimization with Improved Particle Swarm Algorithm

**DOI:** 10.1155/2014/652135

**Published:** 2014-06-12

**Authors:** Mengqi Liu, Miyuan Shan, Juan Wu

**Affiliations:** School of Business Administration, Hunan University, Changsha, Hunan 410082, China

## Abstract

For most enterprises, in order to win the initiative in the fierce competition of market, a key step is to improve their R&D ability to meet the various demands of customers more timely and less costly. This paper discusses the features of multiple R&D environments in large make-to-order enterprises under constrained human resource and budget, and puts forward a multi-project scheduling model during a certain period. Furthermore, we make some improvements to existed particle swarm algorithm and apply the one developed here to the resource-constrained multi-project scheduling model for a simulation experiment. Simultaneously, the feasibility of model and the validity of algorithm are proved in the experiment.

## 1. Introduction

As we all know, large make-to-order enterprises can meet customers' special demands with quick response and relatively low cost. The process is usually composed of three stages: order R&D, sample manufacturing, and batch manufacturing. Whether the profits can be maximized and the requirements of customers on quality and delivery can be met are usually determined at the order R&D stage, during which time the manufacturing ability and the cost composition of the order are informed, and this stage occupies 3/4 of the time needed to finish the order. So it is no doubt that the key competitiveness of large make-to-order enterprises is the order R&D ability. As a result, improving the order R&D ability as well as reducing the cost and shortening the delivery time at R&D stage is crucial to large make-to-order enterprises.

Resource-constrained multi-project scheduling problem is a generalization of the resource-constrained project scheduling problem (RCPSP). And the features of multiple R&D projects in large make-to-order enterprises are as follows: (1) results and timing are very uncertain because the unique characteristic of each project is based upon the degree of innovation; (2) human resource is the main and scarcest resource in R&D projects; (3) the cost of each project is supposed to be as low as possible owing to the limited budget and for the pursuit of expected profits; (4) multi-project R&D environments are dynamic, which originate from technological updating as well as continuous new orders and the change in the priority level of projects; (5) the length of multi-project period will change with customer demands. In order to guarantee that the order with higher priority is delivered on time, the ones with lower priority has to be postponed to release the occupied resource; (6) penalty and rewards exist if the project is postponed or finished in advance respectively, the amounts also should be related to the priority level of projects; (7) the allowed time is limited; (8) working overtime is allowed but should be limited within the scope of labor laws. In this paper, the overtime each day should be less than 2 hours. By the way, overtime wage is 1.5 times higher than the cost of normal working hours. According to the above mentioned features of multiple R&D projects in large make-to-order enterprises and the resource-constrained multi-project model proposed by Wiest and Levy [1], and combined with the improvement made and methods raised by Wiley et al. [2], planned multiple R&D project scheduling model which meets the make-to-order environment is built. However, Wiest and levy only consider the crashing cost and rewards for finishing the project in advance, while the postpone penalty caused by the occupation of other projects has not been considered. Wiley improves the model and also considers the crashing cost, but he also uses the dual code as Wiest and Levy, that is, trimming activity (*i*, *j*) as a project activity between node *i* and node *j*, which is difficult to understand and increases the complexity of the model.

Accordingly, RCPSP has been treated by many approaches. Particle swarm optimization (PSO) is the most common one, which is proposed by Kennedy and Eberhart [3]. PSO is a population based optimization algorithm, and has exhibited many successful applications, ranging from evolving weights and structure for artificial networks [4–6], manufacturing and milling [7], and reactive power and voltage control [8], and estimation to electric power distribution systems [9]. Since particle swarm algorithm is easy to trap into partial optimization and it is difficult to find a solution for multiple problems, many experts have made a lot of improvements to it. Zhang et al. [10] analyze the constrained project scheduling problem by using the original particle swarm algorithm, and then achieve two different scheduling generating methods. And research on the application of particle swarm algorithm to constrained multi-project scheduling problem is attracting more attention than ever. Sha and Hsu [11] apply Tabu search to improve the solution quality. Yin et al. [12] embed a hill-climbing heuristic in the iterations of the PSO. Jiao et al. [13] use the dynamic inertia weight that decreases along with iterations. Liu et al. [14] propose a model in which the center particle is incorporated into the linearly decreasing weight particle swarm optimization (LDWPSO). Unlike other ordinary particles in LDWPSO, the center particle has no explicit velocity and is set to be the center of the swarm at every iteration. Other aspects of the center particle are the same as the ordinary particle. Valls et al. [15] propose a hybrid genetic algorithm (HGA) for the resource-constrained project scheduling problem (RCPSP) with the implication of a local improvement operator and a new way to select the parents. Similarly, based on random keys, Gonçalves et al. [16] present a genetic algorithm for the resource-constrained multi-project scheduling problem, and schedules are conducted by using a heuristic algorithm that builds parameterized active schedules contingent on priorities, delay times, and release dates. Elloumi and Fortemps [17] transform the problem of single objective MRCPSP to a biobjective one to cope with the potential violation of nonrenewable resource constraints and build the fitness function as an adaptive one relying on clustering techniques, aiming to analyze more relevant fitness values. Coelho and Vanhoucke [18] provide a new algorithm, which splits the problem types into mode assignment and single mode project scheduling: the former is solved by a satisfiability (SAT) problem solver and returns a feasible mode selection to the project scheduling step; and the latter is solved by using an efficient metaheuristic procedure to work out the resource-constrained project scheduling problem (RCPSP). Then they execute these two steps in one run with a single priority list, which is different from many traditional metaheuristic methods. Afshar-Nadjafi et al. [19] develop a metaheuristic algorithm, namely, the genetic algorithm (GA), to obtain a global optimum solution or at least a partial one, and then employ the Taguchi experimental design as a statistical optimization technique to calibrate the effective parameters. Furthermore, in the other areas, by the aid of the particle swarm optimization (PSO), optimizing a GA-SVM method [20], predicting single nucleotide polymorphisms (SNPs) and selecting tag SNPs [21] or solving the heating system planning problem [22] are presented. Synthetically, Zhang et al. combine PSO with SVM for classifying magnetic resonance imaging (MRI) brain images [23].

Above all, the improvement of original particle swarm optimization algorithm can be roughly grouped into four categories as follows: (1) the improvement depends on incorporating new coefficients into velocity and position equations of the PSO algorithm or rational selection to the value of coefficients; (2) a significant point of PSO algorithms is to improve the degree of information sharing among the neighbourhoods; (3) the operators of other evolutionary algorithms are combined with PSO's; (4) some mechanisms are designed to increase the diversity of particles in order to prevent premature convergence to local minimum. But the research refers to the application of particle swarm algorithm to resource-constrained multiproject scheduling problem is few. So, in this paper, we perform the part not researched by others, and wield the particle swarm algorithm with some improvements based on the fruit of predecessors.

The rest parts of the paper are organized as follows. In Section 2, we build a resource-constrained scheduling model with multiple projects in large make-to-order enterprises. The goal of minimizing R&D cost should be fulfilled under the condition that the allowed total time of R&D time cannot exceed the limited total time. Next, in Section 3, we put forward the improved dynamic center particle swarm optimization algorithm based on the original particle optimization algorithm. Then the improved algorithm, which includes the equation of the particle and calculation of adaptive values, are presented in Section 4. In Section 5, we change the dual code to single code and make additional adjustments under the given conditions and limitations for meeting the requirements of the model. Then the effectiveness of the model and the algorithm will be proved in the simulation experiment with MATLAB program. And Section 6 is the conclusion.

## 2. Model Formulation

Absorbing the advantages of above literature, as well as considering the problem under more comprehensive conditions, this paper adopts the single code to describe the network of projects and builds a multi-project R&D scheduling model to reschedule projects activities with the minimum cost under constrained resource. The definition of the variables and parameters is as follows.

Assume that the number of projects which need resource distribution is *N*, expressed as *P*_*i*_, *i* = 1,2,…, *N*. The number of activities each project involves is *m*_*i*_. The project activities set is *P*_*ij*_ = (*P*_*i*1_, *P*_*i*2_,…, *P*_*im*_*i*__), *j* = 1,2,…, *m*_*i*_. *P*_*ij*_ means activity *j* of project *i*. The start time of *P*_*ij*_ is *ST*_*ij*_, the finishing time is *FT*_*ij*_, and the immediate predecessor activity set of *P*_*ij*_ is *PS*_*j*_. *A*_*t*_ is the going activity set at time *t*. *T*_*i*_ is the normal working time of project *i*. *T*_*ij*_ is the normal working time of *P*_*ij*_. The crash time of *P*_*ij*_ owing to resource limitations will be expressed as *y*_*ij*_, but it cannot exceed the top level, which is knew as *M*_*ij*_. At the same time, the reward of unit time obtained for finishing the project in advance is *L*_*i*_, and the penalty for postponing is *K*_*i*_. And *T* is the allowed total time of multiple projects, *T* ≤ ∑_*i*=1_^*N*^ *T*_*i*_; TCD is the target completion date. There are two kinds of distributable resource. One is the renewable human resource *R* ^*ρ*^ and the total limitation of it is *R*_*K*_^*ρ*^. The human resource each unit time needed at normal working time of *P*_*ij*_ is *r*_*ijk*_^*ρ*^. When working overtime, the resource remains the same, but the normal working time decreases. Another is the total project budget *R* ^*v*^, which is a continuous variable and nonrenewable resource. The resource budget of each project is *R*_*i*_^*v*^ (*R* ^*v*^ ≤ ∑_*i*=1_^*N*^ *R*_*i*_^*v*^). The cost of unit human resource at unit normal working time is *C*, and the human resource unit time cost because of crashing could be *K*_*r*_, *K*_*r*_ > *L*_*i*_. OH means overhead cost per unit time.

R&D multi-project resource allocation model is as follows:

(1) min⁡ COST=OH×max⁡i⁡max⁡j⁡(FTij−STij)+∑i=1N∑j=1miCrijkρTij+Π1+Π2+∑i=1N∑j=1miKrrijkρyij.

(1) Thereinto,

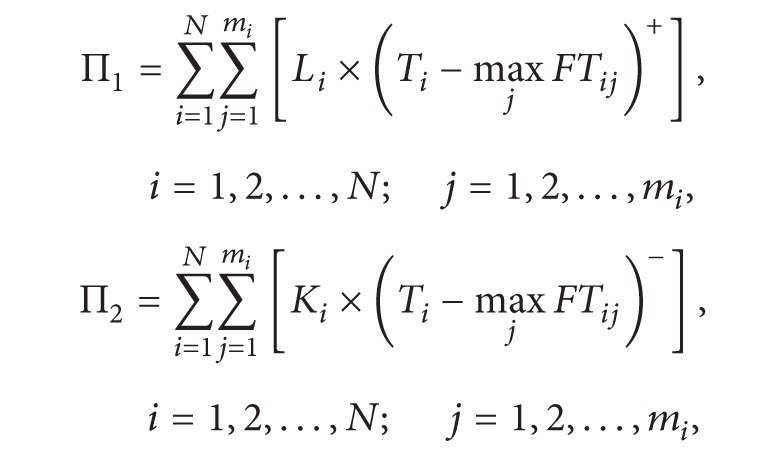
(2)

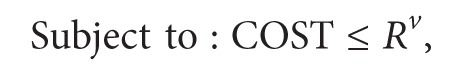
(3)

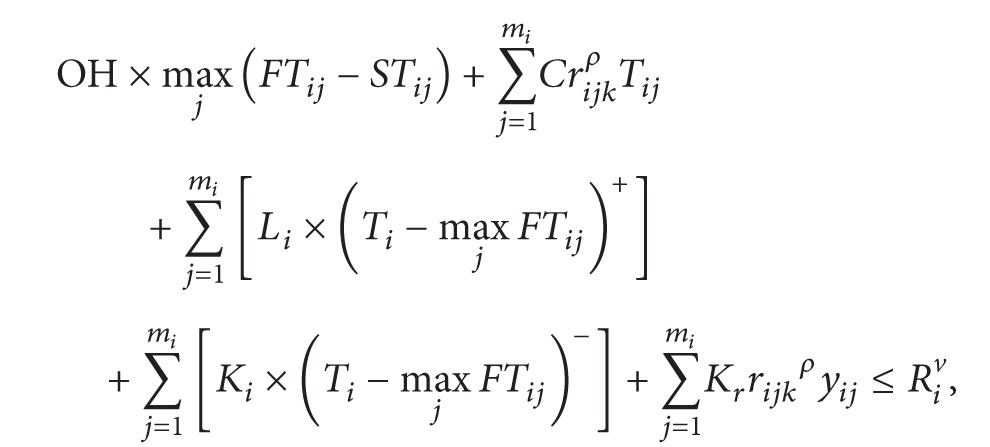
(4)

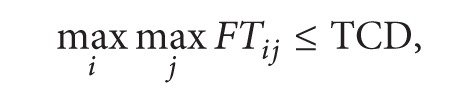
(5)

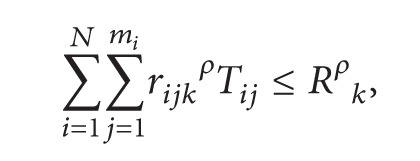
(6)

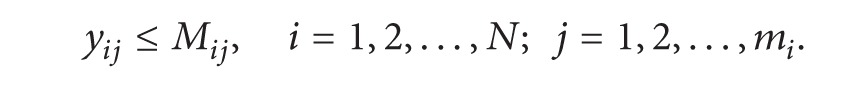
(7)

In the above model, (1) is the target function, meaning the minimum cost of multiple R&D projects. Equation (2) is the rewards for finishing the project in advance and penalty for postponing the project. Restriction (3) means that the total cost of multiple projects must be less than the total project budget. Restriction (4) means that single project is also restricted by its budget. Restriction (5) means that the latest finishing time should be in front of the target completion time. Restriction (6) means the total constraints of human resource in multiple projects. Equation (7) means activities of each project are permitted finished by working overtime, but the crash time must be limited.

## 3. Model Solutions Based on Modified PSO

### 3.1. Particle Swarm Optimization

Each particle is individual and the swarm is composed of particles. The solution space of the problem discussed here is formulated as a search space. Each position in the search space is a correlated solution of the problem. Particles cooperate to find out the best position (the best solution) in the search space (the solution space).

Particle moves toward the best positions *p* and *g* through each iteration, which are found by particles and the swarm respectively. The particles move according to their own velocities, which are randomly generated when approaching *p* and *g*. For each particle *i* and dimension *j*, the velocities and positions of particles can be updated by the following equations:

(8) vit+1=w×vit+c1×rand1()×(pbesti−xit) +c2×rand2()×(gbestt−xit),

(9) $$\begin{array}{c} x_{i}^{t+1}=x_{i}^{t}+v_{i}^{t+1}. \end{array}$$

In (8) and (9), *v*_*i*_^*t*+1^ is the velocity of particle *i* on iteration *t* + 1 and *x*_*i*_^*t*^ is the position of particle *i* on iteration *t*. *pbest*_*i*_ is the best position *p* of particle *i* and *gbest* ^*t*^ is the best position *g* of the swarm on iteration *t*, respectively. The inertia weight *w* was first proposed by Shi and Eberhart [24] and is used to control exploration and exploitation. The particles maintain high velocities with a larger inertia weight factor *w*, and the ones with low velocities have a smaller *w*. A larger *w* can prevent particles and the swarm from being local optima, and a smaller *w* encourages particles to exploit the same search space area. The constants *c*_1_ and *c*_2_ are used to decide whether particles prefer moving toward a best position *p* or *g* severally. And rand_1_() and rand_2_()are random vectors between 0 and 1.

Improvement has been made in light of the defect that GPSO is easily trapped into partial optimization, and a mixed particle swarm optimization algorithm has been proposed. Also the algorithm has been applied to the model of multi-project resource distribution in large make-to-order enterprises.

### 3.2. Dynamic Center Particle Swarm Optimization

After studying the methods that improve original particle swarm optimization algorithm and analyzing the experiment results, a new dynamic center particle swarm optimization algorithm has been proposed. The inertia weight *w* and learning factors *c*_1_, *c*_2_ in the particle velocity equation vary with iterations. The variable range of *w* is [0.4, 0.9]. *c*_1_, *c*_2_ will show linear variation when the iterations increases. At the beginning, *c*_1_ is high, and particle mainly refers to social learning. With the increase of iterations, *c*_2_ goes up and *c*_1_ goes down, and particles come down to cognitive learning. Seeking in partial scope, the values of *c*_1_, *c*_2_ are

(10) $$\begin{array}{c} c_{1}=4\times \frac{\left(\mathrm{Max}\;\text{iteration}-\text{iterations}\right)}{\text{Maxiteration}}, \end{array}$$

(11) $$\begin{array}{c} c_{2}=4\times \frac{\text{iterations}}{\text{Maxiteration}}. \end{array}$$

Meanwhile, a center particle is proposed to visit the center of swarm at each iteration explicitly. After (*N* − 1) particles update their positions under the dynamic PSO algorithm, and their inertia weight and learning factors are varied with every iteration. Then center particle at each iteration is updated according to the following formula:

(12) $$\begin{array}{c} X_{c}^{t+1}=\frac{1}{N-1}\overset{N-1}{\underset{i=1}{\sum }}X_{i}^{t+1}. \end{array}$$

The pseudocode of dynamic PSO is as shown in Algorithm 1.

## 4. Particle Representation and Fitness Evaluation

Generally, It has two methods to represent particles: priority of activities and sequence permutation representation. In this paper, a particle represents a set of activity priorities because it can avoid useless scheduling plan. Actually, in multiple activities, the arrangement is based on the minimum unit activity and assume the immediate predecessor set only contingent on the immediate predecessor relationship expressed by multiple networks, which is the same as that in single scheduling arrangement.

Thus we formulate a particle by the following vector:

(13) $$\begin{array}{c} P=\left(p_{1},p_{2},\ldots ,p_{N}\right),\\ \text{subject}\;\text{to}:\;0\leq p_{i}\leq 1. \end{array}$$

The above particle formulation (13) represented the priority of multiple projects' activities that assure minimum cost. The dynamic center PSO initializes a swarm of particles at random, and then these particles should be moved according to formulation (8)–(12) iteratively. However, the movements of the particles are constrained by adaptive resource bounds.

When particles are updating velocities and positions during iteration, new dynamic center particle swarm algorithm proposed in this paper will be adopted, and the inertia weight and two learning factors are varying too. Meanwhile, the position and velocity of the center particle remain the same.

The swarm intelligence of dynamic center PSO is determined by *p* and *g*. Thus, “fitness” of the particle should be evaluated. We define the fitness function of particle *P* by

(14) fitness(P)=1J(P),

where *J*(*P*) is computed by the objection function (1), using *P* and scheduled with *P*. Thus, the smaller the total cost incurred by *P*, the higher the fitness, and vice versa. The dynamic center PSO enables the particle evolution toward the target with the minimum cost.

In order to evaluate the adaptive values, no matter which particle it is, the elements in the particle must be transferred to a multi-project schedule. There are two ways to generate the schedule: serial scheduling scheme and parallel scheduling scheme [25, 26]. Serial scheduling scheme is arranged based on the task, while parallel scheduling scheme is arranged based on time. Generally, parallel activities of a single project are limited. So to get the optimization of scheduling, it is better to use serial scheduling scheme. But under the multi-project circumstance, there are a lot of parallel activities, so it is suggested to use parallel scheduling scheme to save time. Different from single project parallel generation, in a multi-project environment, priority should be considered in qualified activity set, which means that activities with high priority should be first selected, and then the short term ones will be chosen from them to make the schedule.

To guarantee all the orders placed to large make-to-order enterprises can be delivered in time, each R&D project must be finished before the target completion date. In case of resource constraining the project to be unfinished within normal working time, crashing is needed to shorten the normal working time. In this paper, assume that all staffs have to work overtime when necessary, and then the overworking time can be used to offset normal working time by equal number, which is eight hours per day, namely, one hour overtime means 1/8 of normal working time can be reduced. But the unit cost of crashing is 1.5 times higher than that of normal working time, that is, *K*_*r*_ = 1.5*C*.

## 5. Numerical Analysis

**Figure 1:**
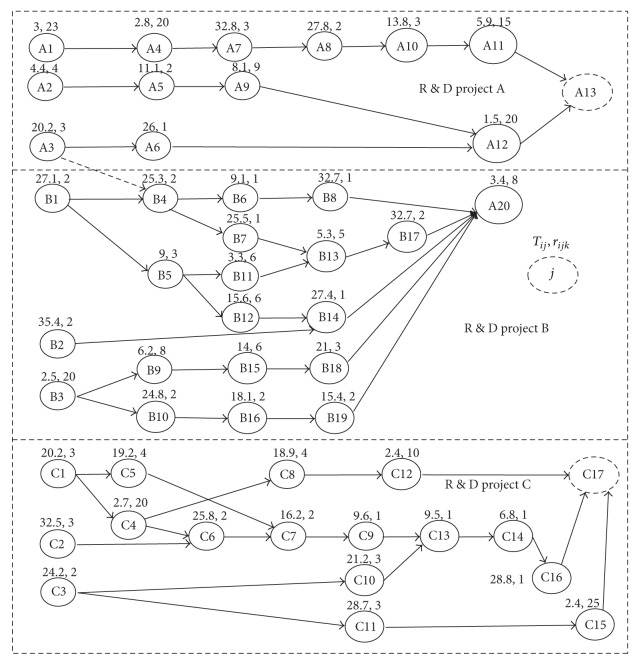
Code network.

To illustrate the performance of the algorithm we proposed, several examples with a variety of datum have been investigated. In this section, dual code network has been changed to single code network as shown in Figure 1. Proper adjustment has been made in accordance with related datum. For example, a dummy has been added to the ends of two parallel activities, which is more suitable to the model. The finishing time of single project is equal to the completion time of the latest one.

Besides, the direct cost has been changed to unit time resource, and *C* = 80, *Kr* = 120, *R*_1_^*v*^ = 11800000, *R*_2_^*v*^ = 16350000, *R*_3_^*v*^ = 1370000, OH = 305000, *R*_*k*_^*ρ*^ = 25.

In the experiment, the maximum iterations of dynamic center particle optimization algorithm is 500, the scale of species group is N = 40, *w* = rand(0.4,0.9), *c*_1_ + *c*_2_ = 4 and varies with iterations in accordance with formulation (10) and (11). After repeated experiments, the results can be obtained: the minimum working time is 78 months, and the minimum cost is RMB 57662330.

## 6. Conclusion

This paper discusses the features of multi-project R&D environments in large make-to-order enterprises with the demand of resource distribution, and makes some improvements to algorithms and models researched before, then puts forward a multi-project scheduling model with crashing cost and postponing punishment. At the same time, based on the analysis of the particle swarm optimization algorithm and its improvement, a new solution to multiple projects scheduling has been arisen. The best solution can be obtained after the experiment to the illustration example through MATLAB program, which proves the correctness of the model we built and the improvements we made in the algorithm.

However, we assume that it has a lot to do to make the study further and deeper, because in this paper, when doing the simulation calculation, the total working time of multiprojects is assumed to be fixed in the first place, and the crashing time will be considered with the methods of permutation and combination. Moreover, the optimal scheme can be achieved only when that all the projects can be finished under constrained resource. Additionally, the uncertainty of demands to resource has not been considered because of the limitation of space and time. All these are worth to be explored in the future.

## Figures and Tables

**Algorithm 1:**